# Clinical Impact of Computational Heart Valve Models

**DOI:** 10.3390/ma15093302

**Published:** 2022-05-05

**Authors:** Milan Toma, Shelly Singh-Gryzbon, Elisabeth Frankini, Zhenglun (Alan) Wei, Ajit P. Yoganathan

**Affiliations:** 1 Department of Osteopathic Manipulative Medicine, New York Institute of Technology College of Osteopathic Medicine, Northern Boulevard, P.O. Box 8000, Old Westbury, NY 11568, USA; efrankin@nyit.edu; 2 Wallace H. Coulter School of Biomedical Engineering, Georgia Institute of Technology, Atlanta, GA 30332, USA; shelly.singh@bme.gatech.edu (S.S.-G.); ajit.yoganathan@bme.gatech.edu (A.P.Y.); 3 Department of Biomedical Engineering, Francis College of Engineering, University of Massachusetts Lowell, Lowell, MA 01854, USA; zhenglunalan_wei@uml.edu

**Keywords:** heart valves, mitral valve, tricuspid valve, aortic valve, pulmonary valve, repair, devices, computational analyses

## Abstract

This paper provides a review of engineering applications and computational methods used to analyze the dynamics of heart valve closures in healthy and diseased states. Computational methods are a cost-effective tool that can be used to evaluate the flow parameters of heart valves. Valve repair and replacement have long-term stability and biocompatibility issues, highlighting the need for a more robust method for resolving valvular disease. For example, while fluid–structure interaction analyses are still scarcely utilized to study aortic valves, computational fluid dynamics is used to assess the effect of different aortic valve morphologies on velocity profiles, flow patterns, helicity, wall shear stress, and oscillatory shear index in the thoracic aorta. It has been analyzed that computational flow dynamic analyses can be integrated with other methods to create a superior, more compatible method of understanding risk and compatibility.

## 1. Introduction

The art of heart valve repairs is constantly developing. Leonardo da Vinci conducted studies in animals and did more than 30 human dissections to accurately interpret the anatomy of fresh specimens and the motion of blood in the beating heart through small metallic tracers. Over half a millennia later, we are still investigating the movement of blood in the beating heart, albeit by means not available to Leonardo. Noteworthy technological improvements [1] have facilitated the evolution of computational methods for heart valve modeling.

The four heart valves include the mitral valve (MV), tricuspid valve (TV), aortic valve (AV), and pulmonary valve (PV). The configurations of the mitral and tricuspid valves are similar, comprising two and three valve leaflets, respectively, with inserted chordae tendineae and anchored to the ventricle walls via papillary muscles. On the other hand, the aortic and pulmonary valves are comprised of three equally sized semilunar cusps or leaflets, which are bound at three commissures. The pressure gradient across each valve controls its opening and closing dynamics. In heart valve disruptions, the design of the valve may be compromised, leading to stenosis (narrowing of the valve) or regurgitation (leakage of the valve). Treatment of these conditions can be surgical, transcatheter, or percutaneous, and include repair or replacement therapies.

Validated using in vitro models, e.g., [2], computational models can be used to (i) aid in the development of diagnostic tools, therapeutic instruments, and innovative prostheses for the treatment of heart valve ailments; (ii) indicate surgical consequences for repair or replacement procedures available for heart valve pathologies, or support pre-procedural planning of appropriate transcatheter or percutaneous therapies; (iii) provide help in medical device regulatory recommendations, and (iv) explain the cause-and-effect associations between cardiovascular biology and hemodynamics [3]. The latter has the prospect to extend our knowledge of disease evolution and progression, thereby allowing the expansion of new translational technologies, diagnoses, devices, and treatment alternatives [4,5].

Computational fluid dynamic (CFD) investigations are a cost-effective mechanism that can be used for the high-resolution evaluation of clinically pertinent flow parameters, e.g., wall shear stress and blood damage. These parameters are of interest during the creation and optimization of manufactured heart valves but are challenging to measure in vivo and/or in vitro. Thus, CFD can be used to augment the understanding gained from clinical and empirical reviews of artificial heart valves. To guide in conducting computational studies of transcatheter heart valve prostheses, a position paper was disseminated by an ISO working group [6], while more recently, the FDA has formulated procedures for evaluating the credibility of computational modeling and simulation in medical device recommendations [7]. Patient-specific modeling has been earning awareness because of its prospect to tailor possible therapies and enhance patient outcomes (e.g., [8,9]). However, there is presently no traditional practice for the patient-specific evaluation of artificial heart valve performance using CFD. Fully patient-specific computational simulations are fairly new and not yet exhaustively validated for a wide spectrum of applications. Similarly, the use of CFD for heart valve modeling is challenged by the intricacy of the interaction between blood flow and the anatomical and/or device configurations concerned, oftentimes necessitating the usage of more costly and convoluted fluid–structure interaction (FSI) models.

This examination emphasizes the role of engineering applications and computational strategies for heart valve modeling, with a focus on the treatment of heart valve conditions. This review summarizes recent computational studies that use various FSI analyses to investigate the heart valves. However, the use of FSI algorithms is still scarce. Hence, recent CFD studies without considering the FSI were also included. Similar review articles on the clinical impact of computational models with different priorities can be found [10,11].

### 1.1. Chordal Repair/Replacement

Degenerative MV disorder frequently leads to leaflet prolapse due to chordal stretching or rupture and resulting in MV regurgitation [12]. As noted before, computational strategies can expand the knowledge attained from empirical methodologies, such as artificial chordae for mitral and tricuspid valve restorations [13]. For example, Toma et al. and Singh-Gryzbon et al. developed a chordal material properties iteration technique which delivered a good match of the computational and experimental coaptation lines between the leaflets in contact when complete closures are reached in the mitral [14] and tricuspid [15] valves, respectively. Watton et al. used the immersed boundary (IB) approach to simulate a chorded prosthetic MV, lodged in a cylindrical conduit, subject to a physiological recurring fluid flow [16]. While it is inferred that the use of artificial chordae implantation is superior in a range of pathological environments, several issues remain in their usage, particularly the appropriate judgment of their length [17,18]. Computational procedures that unravel these points may enhance patient outcomes. In [19], the resulting regurgitation from 51 distinct potential ruptures in a single-subject subvalvular apparatus is demonstrated. Failure of MV reconstructive strategies usually may be elucidated to extreme or progressive alterations of subvalvular apparatus [20]. As articulated in [21], while the importance of keeping the integrity of papillary muscle, chordae tendineae, and MV cuspid is clear, the knowledge of the highest resistance that a primary tendinea chorda can resist is not known. An examination of the not-so-recent literature on recurrent mitral regurgitation due to ruptured synthetic chordae is delivered in [22]. More current chordal cutting/rupture investigations, and studies on the significance of preserving the MV apparatus, can be located [23,24,25,26,27,28,29,30]. A comparative analysis evaluated the medium-term outcomes of the loop procedure in comparison with the widely embraced leaflet resection approach for the restoration of isolated posterior mitral leaflet prolapse [31]. A comparison of survival aspects of MV repair versus prosthetic substitute for degenerative diseases during twenty years was completed demonstrating long-term data to defend the merit of restoration versus prosthetic valve replacement [32]. In patients with degenerative MV and ischemic heart conditions, MV repair grants a survival benefit over the substitute that becomes apparent about two years after the procedure [33]. When pursuing a higher benchmark for degenerative MV repair, evaluating the durability of MV repair is integral [34]. Long-term consequences of MV repair with chordal replacement were reviewed [35,36,37]. Nonetheless, the concern of how to safely estimate the neochordae length remains [17,18,38,39]. For that cause, techniques are devised to perform beating-heart implantation and off-pump adjustment of neochordal length [40]. Additional examination of enlarged hearts and papillary muscle displacement is essential to retain the complete range of pathologies. Anchoring of neochordae at the papillary muscles, thereby imitating the authentic anatomy, should be favored over the left ventricular apex [41]. In patients with chronic functional ischemic mitral regurgitation, papillary muscle relocation has the prospect to yield reverse left ventricular remodeling. Stitches connected between epicardial discs and individual trigones can be utilized for papillary muscle relocation [42,43]. The papillary muscle relocation strategies are seen to dramatically benefit ischemic patients by impacting the left ventricular form and operation more efficiently compared with the full retention of the mitral subvalvular apparatus if the MV is to be replaced [44]. Some methods are investigated independently versus in combination with other techniques. For instance, in patients with ischemic mitral regurgitation, combined MV repair and revascularization resulted in comparable five-year survival when compared with revascularization alone. Regardless, combined MV repair and revascularization generated less postoperative mitral regurgitation [45].

### 1.2. Heart Valve Repairs/Devices

Numerous studies have been executed to investigate the impact of medical devices on decreasing the stress in diverse MV regions [46,47,48]. While the physio ring is regarded as an improved rendition of the traditional rigid ring, and the physio ring is more widely employed, long-term results of repair for degenerative MV disease with the classic and physio rings are equivalent [48]. However, the low incidence of reoperation and late cardiac events indicates that the physio ring, with its intrinsic flexibility, presents an indisputable benefit in the application of remodeling strategies in MV reconstruction [49]. Some therapies of choice for chronic ischemic mitral regurgitation annul active annular movement and immobilize the posterior leaflet. In a model of chronic ischemic mitral regurgitation, septal–lateral annular cinching sought to uphold regular annular and leaflet dynamics was tested [50]. A decrease of the annulus with an undersized ring has once seemed to be the select surgical choice to rectify ischemic mitral incompetence [51,52]. Nonetheless, numerous investigations uncovered substantial residual and repeat rates of mild to severe mitral incompetence in 30% of patients within 6 months of surgery [53,54,55,56,57]. Mitral valve replacement strategies in patients with left ventricular dysfunction are often chaperoned with other techniques for more promising left ventricular remodeling compared with total retention of the mitral subvalvular apparatus during MV replacement [58]. Surgical repair is the most routine procedure used to rectify mitral regurgitation. However, the effectiveness of other techniques is still examined. The efficacy of a procedure is specified using an immense combination of factors, such as the durability of the repaired valve as well as the valve’s function and hemodynamics under stress states. Thus, a myriad of studies are carried out to assess these parameters at follow-ups [59,60,61,62]. By approximating edge-to-edge repair (to repair ruptured/elongated chords) with chordal replacement, it was discovered that edge-to-edge repair and chordal replacement are sufficiently suited for the restoration of both the anterior and posterior leaflets [63]. Regardless, among patients experiencing transcatheter MV edge-to-edge repair with the MitraClip device, a pertinent ratio (2–6%) requires open MV surgery within 1 year after unsuccessful clip implantation [64]. Both in vivo and in silico examinations evaluated the combined force transfer from the papillary muscle tips to the MV via the chordae tendineae, and thereby quantified the force shared through the papillary–chordal complex to augment left ventricular ejection [65,66].

### 1.3. Repair versus Replacement

The outcomes underscore the significance of early detection and review of mitral regurgitation [67]. The most satisfactory short-term and long-term results are gained in asymptomatic patients worked on in state-of-the-art repair centers with low operative mortality and high repair rates [68]. The durability of a successful mitral reconstruction for degenerative MV condition is not consistent, and this should be accepted when asymptomatic patients are proposed early MV repair [69]. Early diagnosis and surgery are paramount as a life-saving standard for infants with acute MV chordal rupture [61,70]. The unique vision of staging of the valvular diseases, newer predictors, and controversy of “watchful waiting” versus “early surgical intervention” for severe, asymptomatic, primary mitral regurgitation are examined in a study that outlines the current interpretation of primary, degenerative mitral regurgitation concerning etiology, complete examination, natural history, and control [71]. Based upon a sounder knowledge of the natural history of mitral regurgitation, the unsatisfactory effects of medical therapies, the adverse consequence of anomalous left ventricular dimensions and function, and manifestations of long-term survival, a directive presently exists for early surgical repair of mitral regurgitation before the start of symptoms and considerable left ventricular dysfunction [72]. New valve pathology after a repair oftentimes results in recurrent mitral regurgitation. Successive mitral re-repair is conducted in nearly half of patients and is associated with outstanding survival, enhanced ejection fraction, and more significant regression in ventricular proportions compared with valve replacement [73,74]. However, an observational analysis found that MV repair in coronary artery bypass grafting patients with ischaemic mitral regurgitation and depressed left ventricular ejection fraction is not incomparable to mitral valve replacement concerning operative early mortality and mid-term survival [75]. A meta-analysis of randomized controlled trials and adjusted observational studies demonstrated that for patients with ischemic mitral regurgitation, MV repair seems to be unassociated with a noteworthy reduction in both early and late all-cause mortality compared with MV replacement [76]. The mechanisms of MV repair failure as well as aspects that meaningfully impact the probability of a successful re-repair can be located in [77]. When comparing MV re-repair versus replacement following failed initial repair, it was uncovered that they are associated with comparable postoperative outcomes [78]. Repair of rheumatic MVs has been met with narrow success. Due to residual diseased leaflet tissue, the hemodynamic obstruction continually endures after repair. An assertive strategy to rheumatic MV repair with extreme excision of the diseased leaflets area, and subvalvular apparatus and subsequent reconstruction, intending to extract all diseased valvular tissue, was devised and executed [79]. Data comparing processes of MV repair and replacement for ischemic mitral regurgitation are primarily restricted to small, non-randomized retrospective trials [80]. The only randomized trial data to investigate this topic indicated no distinction in mortality with either replacement or repair; however, the replacement was shown to be invariably associated with higher rates of mitral regurgitation recurrence [80]. Regardless, the use of replacement heart valves persists to grow due to the raised preponderance of valvular heart disorders resulting from an aging population [81].

### 1.4. Tissue Engineering

Traditional replacement treatments for heart valve disorders are associated with considerable deficiencies. Heart valve diseases harbor a significant chance of morbidity and mortality. Results are particularly enhanced by valve replacement, but presently available mechanical and biological replacement valves are associated with difficulties of their own. Mechanical valves have a high rate of thromboembolism and demand lifelong anticoagulation. Biological prosthetic valves retain a considerably shorter lifespan, and they are inclined to degradation and ripping. Both types of valves lack the ability to grow, making them specifically troublesome in pediatric patients. Scientific and technological breakthroughs through the last 50 years have put forward diverse surgical options to patients with progressive heart failure encompassing surgical ventricular repair to surgical gene therapy and stem cell replacement of the diseased ventricles [82]. The specialization of tissue engineering has appeared as a compelling option in the quest for improved heart valve replacement designs. One of the tenets behind this vision is the transplantation of living elements, implanted in a suitable scaffold fabric, to the diseased area where the structure becomes merged with patients’ tissue to revive natural function [83]. There are various 3D printing procedures that rely on the types of materials employed. Various types of organs (bone, cartilage, heart valve, liver, and skin) assisted by 3D printed scaffolds and printing methods that are applied in the biomedical specializations were examined [84]. Flanagan and Pandit assembled a review of the advancement that has been made in the evolution of living manufactured heart valve options [85]. Some tissue-engineered heart valves have had clinical success, whereas others have failed, with structural deterioration resulting in patients’ deaths. Blum et al. discussed the need for tissue-engineered heart valves to treat pediatric patients with valve diseases, the history of tissue-engineered heart valves, and a future that would aid from the extension of the reverse translational trend in this area to retain small animal investigations [86]. Regardless, heart valve tissue engineering suffers from narrow long-term performance in vivo because of unbridled tissue remodeling phenomena, such as valve leaflet shortening, which often yields valve failure regardless of the bioengineering procedure employed to generate the implant [87]. The integration of computationally inspired heart valve configurations into tissue engineering procedures could steer tissue remodeling toward long-term functionality in tissue-engineered heart valves [88].

### 1.5. Imaging Modalities

The European Association of Echocardiography in collaboration with the American Society of Echocardiography has devised the guidance for the use of echocardiography in new transcatheter interventions for valvular heart disorders [89]. The use of echocardiography for catheter-based therapies is paramount for the success of the procedures [90]. Nevertheless, to evaluate the effective orifice area, it was discovered that the results are undervalued when using the 2D transesophageal echocardiography approach compared with the 3D methods (multislice CT, MRI and 3D transesophageal echocardiography) [91,92]. Using 3D echocardiography simultaneously with geometric modeling and rendering strategies, high-resolution, quantitative, 3D procedures for imaging the human MV are created [93]. Tamborini et al. conducted a comparison between different 3D echocardiographic rendering devices in the imaging of percutaneous edge-to-edge MV repair [94]. Echocardiography can determine MV features that are predictive of successful valve repair. However, even with echocardiography specifying MV attributes, repair in hypertrophic cardiomyopathy patients with symptomatic obstruction who experience myectomy, although long-lasting, is achievable in only about half of patients [95]. A multi-center analysis discovered that software modeling utilizing pre-procedural computed tomography angiography is a detailed methodology for indicating the risk of mild and severe mitral regurgitation due to paravalvular leak after transcatheter MV replacement [96]. The function of cardiac computed tomography for assessing the MV has been restricted since echocardiography is the primary form of evaluation. Yet, recent advances in cardiac computed tomography have facilitated thorough evaluation of the anatomy and geometry of the MV [97].

### 1.6. Transcatheter Repairs

Percutaneous intervention for MV disorder has been designated as an alternative to open surgical repair, especially in high-risk and inoperable candidates [98]. With procedures completed earlier in disease advancement and improved patient longevity, the demand for a repeat intervention is not rare. With the associated dangers of reoperation and patient comorbidities, percutaneous procedures for acute or delayed failure after ring annuloplasty are arising [99]. A myriad of catheter-based techniques for patients with regurgitant as well as stenotic valvular disease is presently at disposal. A thorough understanding of mitral valvular anatomy is vital for the selection of patients, the implementation of devices, and further improvements of these transcatheter practices if they are ultimately to deliver procedural and clinical triumph [100,101]. For mitral stenosis employing either a single balloon or double-balloon procedure, percutaneous MV dilatation is routinely executed. Computational approaches are utilized to compare the two techniques [102]. A screening algorithm to evaluate anatomical eligibility for transcatheter MV replacement in patients with severe mitral regurgitation, based on simple multislice computed tomography measures was designed [103]. The existing overall range of interventional treatment options enables patient-oriented therapies individually targeting various mitral regurgitation pathologies. The current variety of transcatheter treatments for relevant mitral regurgitation is debated in [104]. Similarly, new transcatheter procedures to execute tricuspid annuloplasty are unwinding and are presented in the clinical practice. Nonetheless, clinical experience is limited [105]. Furthermore, present refinements in transcatheter valvular interventions have resulted in a growing market for refined cardiac imaging to help conduct these operations [106]. A summary of fundamental notions linking to transcatheter MV replacement pre-procedural planning, with distinct priority on imaging-based techniques for indicating transcatheter MV replacement-related left ventricular outflow tract obstruction can be located in [107,108].

## 2. Computational Simulations

To determine the coupling between the fluid and structural domains, FSI strategies are utilized. Particularly in computational simulations meant to imitate the functions inside the human body, intricate dynamics are present, e.g., heart valves opening and closing every second interacting with blood flow. Consequently, for physiologically authentic simulations, the fluid dynamics associated with the valves, the structural mechanics of the valves, and tissue characteristics, should be modeled concurrently. Nonetheless, standard FSI studies present several challenges, e.g., considerable extra computation time.

FSI simulations can be separated into three significant classifications: (1) Pseudo-state simulations are generally used to investigate the downstream flow domains of heart valves under the supposition that the valve is unmoving, and they can be modeled utilizing ordinary computational fluid dynamics strategies for flow fields [109]. (2) One-way FSI lets heart valves move under a stipulated geometric deformation. The prescribed structure dynamic movement impacts the fluid flow but not contrariwise. In two-way FSI (3), the most demanding type of FSI simulation, the structural and fluid fields influence one another. The structural model of a two-way FSI solver requires adequately representing material properties and the interaction between the leaflets and the surrounding fluid. Naturally, most two-way FSI solvers can solve one-way FSI problems.

**Figure 1:**
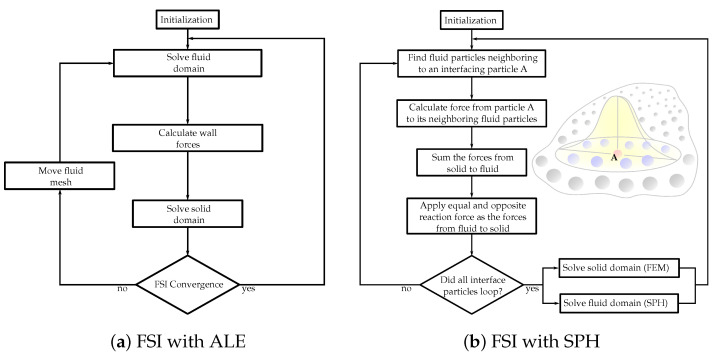
Flowcharts of the FSI solution algorithms with (**a**) arbitrary Lagrangian–Eulerian (ALE) and (**b**) smoothed-particle hydrodynamics (SPH) methods.

Two techniques are utilized for the coupling between the fluid and structure domains. (A) Partitioned technique: The fluid and solid domains are treated individually with two separate solvers (Figure 1a). Communication between the two solvers is passed along their domain interface. Since each domain is solved employing a different solver, autonomous numerical algorithms can be involved to solve the fluid and solid equations. Consequently, less memory storage is demanded compared to the monolithic approach. However, in the FSI heart valves simulations, which typically include large deformations, this technique tends to face converge issues due to stability problems [109]. (B) Monolithic approach: The fluid and structural domains are solved simultaneously by discretizing the problem into a single system of equations employing a single numerical algorithm [110]. This generates fewer convergence issues since the joint impact of the two domains on one another is incorporated directly. However, for extensive 3D problems, with a high number of degrees of freedom, a prohibitive quantity of memory storage is required.

An alternative method to classify FSI techniques is to (1) body-fitted and (2) non-body-fitted methods. This categorization depends on whether the computational fluid domain mesh conforms to the borders of the computational solid domain mesh. The Arbitrary Lagrangian–Eulerian (ALE) approach is an illustration of a body-fitted method, and the Immersed-Boundary (IB) method is one of the non-body-fitted methods. The IB approach is an efficient way of modeling fluid–structure interactions. Numerical simulations employing coupled MV and left ventricle models are devised utilizing IB and finite element methods (FEM) [111]. An FSI model of the left atrium and MV employing an IB-FEM framework is utilized in [112] to examine the impacts of diverse pathological conditions. Regardless, it bears two major constraints: namely, the difficulty of use and capacity to model static loading. Additionally, one other thing can be detected in all the IB analyses, i.e., 3D models employed seem to be geometrically streamlined with the purpose of evading computational instability and convergence problems.

Thus far, the ALE approach is the most traditional technique embraced in industrial applications. This conforming mesh method divides the computational domains associated with the structure and fluid. Considering the extensive deformation of the heart valve structures together with the connection between the fluid and solid elements, it demands mesh adaptations for the fluid domain, which significantly diminishes computational efficiency and results in poor mesh quality. Since remeshing is essential, it may result in artificial diffusivity and instabilities. The IB method embeds the structure to the static fluid mesh implicitly, which delivers a significant benefit for simulating largely moving/morphing structures. Nevertheless, the near-wall flow resolution of the leaflets of the IB approach may be inadequate to the ALE method.

Peskin et al., in 1997, presented FSI simulations in prosthetic and biological heart valve models with the muscular heart wall retained. Their models depicted the capacity to apply Navier–Stokes equations to moving solid immersed boundaries [113]. In 2003, Tang et al. employed a 3D thick-wall model to imitate blood flow in the carotid arteries and presented asymmetric stenosis to quantify the impact of stenosis while mimicking the pressure conditions on blood flow and artery contraction [114]. This strategy was then expanded upon, including geometries reconstructed from CT scans well resembling the intricate anatomy of the human artery [115,116]. The usage of traditional mesh-based numerical procedures for biomedical applications remains a challenge, and it is nevertheless the standard approach to streamline the computational models by skipping the fluid domain [117]. However, lately, studies can be encountered demonstrating the benefit of smoothed-particle hydrodynamics (SPH) techniques, as shown in Figure 1b, for accurately executing simulations even within the context of blood flow and thrombosis [118]. A more thorough overview of FSI algorithms employed to simulate heart valves can be found in [119].

The intricacy of computational simulations that involve heart valves (e.g., complex geometries and large deformations) makes SPH well suited to execute these FSI calculations, namely SPH methods mixed with high-order FEM. Employing SPH methods brings numerical stability because the communication between the solid and fluid domains is fairly straightforward to treat numerically. Moreover, it is more manageable to parallelize SPH. Consequently, it is achievable to run FSI simulations with convoluted geometries, i.e., conserving all their geometrical details; and, at the same time, maintaining the simulations numerically steady, accurate, and parallelized on a standard GPU workstation. Thus, the user can run these complicated simulations “under the table” rather than on large supercomputers, with the typical runtime being only hours/days as opposed to weeks/months.

**Figure 2:**
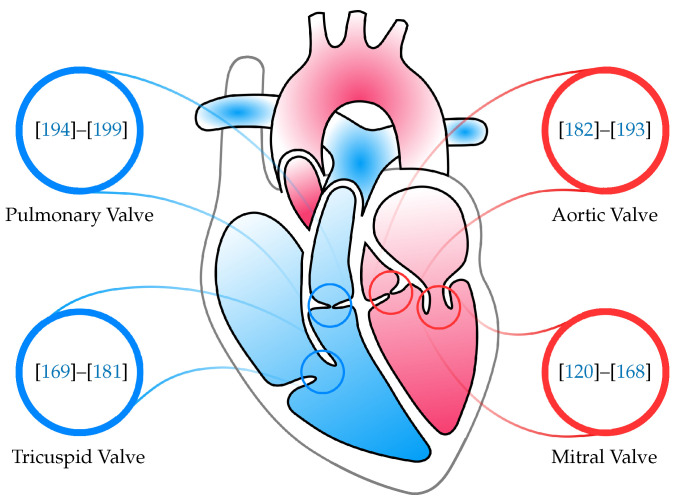
Organization of this review paper. The references are further divided according to which of the four heart valves is their main focus.

The following literature cited is divided according to the four heart valves (Figure 2).

## 3. Mitral Valve

Computational hemodynamic simulations employing conventional numerical strategies are conducted to apprehend the impact of MV leaflets on blood flow [120,121]. Similarly, the vortex formation process inside the left ventricle is investigated concerning the dynamics of the mitral leaflets while they interact with the flow crossing the valve during diastole [122,123,124,125]. A computational analysis demonstrated that the presence of the MV and the shape of its leaflets significantly qualifies the building and development of vortex structures in the left ventricle [126]. Similarly, computational techniques are employed to evaluate MV leaflet in-plane strains from clinical images [127], while other investigations concentrate on the MV chordae, e.g., to quantify their load-dependent adaptations in the collagen fiber architecture for the strut chordae tendineae-leaflet insertion [128].

The intricacy of heart valve geometries, mixed with the extensive deformations they experience with every heartbeat between their fully opened and closed positions, make SPH well suited for running FSI computations. The SPH approach was illustrated and validated in several reports on MV closure [14,66,129,130]. Henceforward, it was utilized to evaluate several diseased MV states [19,131,132,133] and applications of medical devices developed to rectify them [46,134,135,136]. In addition to the MV, other valves have been researched using the same procedures [15,130,137,138,139,140]. The SPH technique has been validated to investigate the hemodynamics of the left ventricle [141]. Interaction between bioprosthetic heart valves and blood flow was alike analyzed employing SPH [142].

Chordal transposition is employed in MV repair [143], yet the impacts of second-order chordae transection on valve operation have not been broadly investigated. In vitro experimentations utilizing excised porcine valves indicate that second-order chordae may intercede leaflet tethering in the setting of apical displacement of papillary muscles, as might be witnessed in patients with ischemic mitral regurgitation [144]. Occasional investigations assessed leaflet coaptation, 3D anterior MV leaflet shape, and valve competence after clipping anterior second-order chordae in vivo [27] and in silico [19]. In vitro examinations using stress–strain analysis on excised porcine mitral chordae by Kunzelman and Cochran [145] have ascertained that primary (first-order) chordae are considerably more inflexible (with higher stress at any given degree of strain) than second-order chordae. Accordingly, it was hypothesized that due to their number and mechanical properties, primary chordae endure the prevalence of systolic pressure load exerted on the mitral leaflets. For instance, in [19], 51 potential chordal ruptures on a single MV were examined. A primary chord (one with a large diameter) was clipped in two distinct locations. Yet, one cut resulted in considerable regurgitation, and the other cut (on the same chord) did not particularly transform the regurgitant orifice area. In that singular location, a large number of second-order chords averted the leaflet prolapse.

During transcatheter MV implantation, encroachment on the left ventricular outflow tract may generate a flow obstruction. Therefore, the proper placement and dimensions of mitral prostheses in transcatheter MV implantation is vital [108]. Patient-specific CFD simulations of transcatheter MV implantation with various cardiac anatomy and insertion inclinations were conducted to anticipate the consequence of transcatheter MV implantation exploiting image-based computational models [146]. The quantification of structural and hemodynamic variables by computational modeling may foster more accurate prognoses of the left ventricular outflow tract obstruction in transcatheter MV replacement, especially for patients who are evaluated to bear a marginal risk of obstruction [147].

Image-based defined displacement can be executed to use the patient-specific shifting of the ventricle in the computational MV investigations. In [148], their pipeline contains image processing of the left ventricle and the MV and numerical examination of cardiac hemodynamics in a moving domain with image-based specified displacement. Patient-specific geometry and activity of the left ventricle are evaluated employing the ALE strategy, while the reconstructed MV is engrossed in the computational domain utilizing a resistive method. Hemodynamic testing employing 3D printing and CFD preoperatively may deliver more additional data in mitral repair than a conventional image dataset [149]. Computational investigations supply a visualization of flow patterns (in both long- and short-axes), which then can be quantified with flow analyses. It was discovered that in comparison to a native valve, valve implantation boosted the balance of the mitral inflow staying in the basal area and consequently increased the residual volume in the apical region [150]. Computational models can additionally be employed to optimize the treatment chances; e.g., FEM is used to compare several indirect mitral annuloplasty percutaneous restoration practices to determine the least-invasive remedies for a considerable inoperable patient population [151].

Nevertheless, whether stress distributions from these computational models translate into concrete and applicable intraoperative conclusions is debatable. For instance, a mere slight modification in the neochordae location or length could seriously transform the leaflet action and stress allocation in the MV computational model. It is irrational to anticipate that such diminutive adaptations could be executed by a surgeon operating in a restricted field of view [152]. While computational modeling of the MV is a hopeful path to enhance the surgical outcomes, the intricate MV geometry thwarts the usage of simplified models. In addition, the absence of comprehensive in vivo geometric data raises considerable challenges in the evolution of patient-specific computational models [153]. Nonetheless, geometry does play a major role in MV mechanics and thus highly affects the precision of computational models emulating MV function and repair [154]. While it is widely debated that conserving the intricacy of the complete mitral apparatus is essential for attaining practical results computationally [155,156,157], there is likewise demand for fast image-based MV simulations employing individualized semi-automatically produced computational models of MV geometries [158]. Despite the unprecedented advancement in artificial intelligence, numerical algorithms, computer capacity, and data (and its increasing effect on industrial/monetary refinement), the evolution of computational models that can be solved with lower computational endeavor is always reasonable and unremitting [159,160]. For instance, a semi-automated framework that incorporates machine learning image analysis with geometrical and biomechanical models to create a patient-specific MV manifestation that integrates image-derived material properties is formed [161]. It is worth mentioning that when extracting an MV geometry from the identical set of medical images, considerable discrepancies can be encountered when segmented by distinct users [162,163]. The sensitivity of MV model execution to the precision of the input geometry is addressed [164]. Three distinct chordae models, namely elaborate, ‘pseudo-fiber’, and simplified chordae, are compared to resolve how diverse chordae representatives influence the dynamics of the MV [165]. Investigations on the inter-user variability of landmarks in MV segmentation deduce that errors delivered as a result of the user dependency were comparable to the deviations of computed hemodynamics [166]. A review outlining the state-of-the-art modeling of the MV, including stationary and dynamics models, models with FSI, and models with the left ventricle interaction, can be located in [167]. A summary of pertinent MV conditions, and the development of numerical models of surgical valve repair strategies, can likewise be found in [168].

## 4. Tricuspid Valve

Tricuspid regurgitation is an ordinary discovery present in a considerable number of asymptomatic patients. However, mild or more harmful tricuspid regurgitation is associated with a poor prognosis [169]. The investigations highlight that the spectrum of TV disorders is beyond that of the annulus and the leaflets [170,171]. There is no class I indication for surgical remedy of isolated functional tricuspid regurgitation in the existing policies [172,173]. On the grounds that right heart failure and pulmonary hypertension are typical disorders in these patients [174], intrahospital mortality after isolated tricuspid surgery is around 10% [175]. Mechanically induced transformations in the TV extracellular matrix structural elements, e.g., collagen fiber spread and dispersal (to resolve the overall macro-scale tissue reactions and thereafter its function/malfunction in physiological/pathophysiological states) are quantified in [176]. Computational modeling can be employed to enhance the interpretation of TV biomechanics and addendum understanding attained from bench-top and large animal investigations. A computational model of the TV, using high-resolution micro-CT imaging and FSI simulations, was produced by Singh-Gryzbon et al. [15]. A computational multi-scale procedure was employed to analyze mechanically induced transformations in TV anterior leaflet microstructure [177]. The computational investigation concerning TVs is presently inadequate, and most analyses still concentrate on the development of structural models rather than engaging FSI calculations. For instance, three FEMs were assembled from human subjects with healthy TVs from CT images incorporating detailed leaflet geometries, realistic nonlinear anisotropic hyperelastic material properties of human TV, and physiological boundary conditions tracked from CT images [178]. As of late, an FEM of one porcine TV geometry was formed to study how diverse pathological disorders impact the general biomechanical function of the TV [179]. There were three immediate observations from that examination. Firstly, the outcomes of the papillary muscle displacement revealed more prominent inconstancies in the TV biomechanical function. Secondly, compared to uniform annulus dilation, the nonuniform dilation rendered more apparent differences in the stresses and strains for the three TV leaflets. Finally, outcomes of pulmonary hypertension exhibited opposing tendencies compared to the papillary muscle displacement and annulus dilation scenarios.

Some of the facts that have been fathered during the development and investigation of MV devices can also be applied to the TV. An in-depth understanding of the bizarre anatomy of the TV and of the right heart chambers, with disparities and similitudes between the two atrioventricular valves, is essential to overcoming the characteristic challenges connected to transcatheter TV treatments [180,181].

## 5. Aortic Valve

Even with the current improvements in computer technology, and numerical algorithms, that permit the inclusion of complex truly patient-specific geometries together with FSI strategies, designing simpler (e.g., structural only) analysis techniques for the simulation of AV closure will always be in demand [182]. An outline of numerical approaches for FSI models of AVs can be located in [183]. A more current assessment of general computational strategies for the aortic heart valve and its replacements can be encountered in [184].

A string of large eddy simulations validated by particle image velocimetry was completed on physiologically representative aortic stenosis models to systematically depict the blood flow in mild, moderate, and severe aortic stenoses [185]. Employing two patient-specific aortas diagnosed to carry pathological dilation of the ascending segment, a computational hemodynamics approach was devised to examine how the morphotype and the functional state of AV would impact the attributes of blood flow in aortas with pathological dilation, particularly the intensity and diffusion of flow turbulence [186]. The postoperative ventricular hemodynamics of substituting both aortic and MVs are not sufficiently comprehended. A computational FSI analysis was employed to generate an improved interpretation of this outcome by modeling a left ventricle with the aortic and MVs substituted with bioprostheses [187]. Other, more superficial, numerical investigations of patient-specific left ventricular models with both mitral and AVs using FSI calculations can be encountered [188,189].

Functional 3D modeling has developed to incorporate a hemodynamically relevant AV model, where multi-material 3D printing was employed to beget patient-specific functional models that were then validated utilizing Gorlin catheter-based and Doppler continuity-based techniques [190]. A proof-of-concept analysis integrating 3D FSI models with idealized geometries indicates that there are distinct discrepancies across haemodynamics and valve mechanics associated with bicuspid AV phenotypes, which may be essential to successive functions associated with their pathophysiology processes [191]. The distinctions in hemodynamics and mechanical properties of bicuspid AVs with various phenotypes throughout the cardiac cycle using FSI calculations were discovered [192]. The conclusions of that investigation imply distinctive contrasts in the hemodynamic characteristics and valve mechanics of different bicuspid AV phenotypes, including various severity of stenosis, flow patterns, and leaflet strain, which may be vital for the prognosis of other ensuing aortic disorders and differential treatment plan for specific bicuspid AV phenotype. While FSI calculations are yet barely employed to study AVs, CFD is utilized to evaluate the impact of various AV morphologies on velocity profiles, flow patterns, helicity, wall shear stress, and oscillatory shear index in the thoracic aorta [193].

## 6. Pulmonary Valve

The PV holds an equally consequential function in the circulatory system. However, there are remarkably few mathematical models to accurately emulate its function. Some models were devised employing simplified geometries and without accounting for the FSI [194]. A pilot study sought to prove the feasibility of reconstructing right ventricle action and simulating intracardiac flow in corrected tetralogy of Fallot patients, solely employing traditional cardiac MRI and an IB approach [195]. The impact of a percutaneous PV reducer on hemodynamics in dilated right ventricle outflow tract is analyzed by computational modeling [196]. Similarly, a reduced-order computational technique was presented as an efficient design analysis of a reducer stent to be percutaneously implanted in dilated right ventricular outflow tracts [197]. Since local shear stress and pressure are predictive for intimal hyperplasia and wall deterioration, CFD computation was employed to select the optimal degree of oversizing for a 12 mm native right ventricle outflow tract with the expectancy that the local hemodynamics may clarify intimal hyperplasia [198]. Likewise, to uncover the impacts of conduit oversizing on the hemodynamics observed after conduit implantation and outgrowth, three different sizes of valved conduits, including the largest possible conduit size, virtually implanted in a child-sized healthy pulmonary artery and the related adult-sized model were studied employing CFD [199].

## 7. Discussion

Valvular heart disorders are a prominent health burden [200]. The remedies for such conditions depend on medication, valve restoration, and replacement with artificial (mechanical and bioprosthetic) heart valves. However, as outlined, long-term stability and biocompatibility issues are conveyed, emphasizing the demand for developing more long-lasting and efficacious replacements [201]. They may assist in the evolution of unexplored or improved diagnostic mechanisms and therapeutic devices, and they can help anticipate patient outcomes. Computational procedures that unravel issues, such as flow patterns, wall stress, and anatomic eligibility, may enhance valve replacement patient outcomes.

Many computational investigations sidestep employing FSI computations. Computational fluid dynamics is still more standard. Simplified open-source software frameworks for cardiovascular integrated modeling and simulation are devised [202]. The purpose is to concoct a software environment that delivers powerful computational hemodynamics tools accessible to a broad audience. To attain that goal, model sharing and reproducibility examinations in scholarly publishing are urged to improve the quality of modeling and simulation analyses, which would also inform future users of computational models [203].

It is reasoned that computational simulations of heart valve closures ought to be patient-specific for them to be practical. To maintain patient-specificity, they need to employ geometries and boundary/initial conditions without simplifications and to retain the FSI calculations. Computational simulations bear the prospect of the predictive capability to determine if a valve would more assumably benefit from restoration or replacement. The mixture of smoothed-particle hydrodynamics and the high-order finite element method delivers the capacity to maintain the calculations integrity while minimizing needed runtime on the GPU workstations. Nevertheless, many simulations remain somewhat costly within a clinical context. In addition, the inevitable inter-user variability appears to be partially accountable for errors in calculated hemodynamics, specifically in complex valve geometries, such as the MV. Furthermore, the employment of adequately validated models is vital [204]. Similarly, just like detailed patient-specific geometries, proper realistic boundary and initial conditions need to be utilized to preserve the authenticity of the simulations. There are processes occurring simultaneously on molecular, cellular, and organ levels. Multiscaled computer models are developed to incorporate these levels into a single model. For example, Campbell et al. outlined an approach to create patient-specific computer models that integrate genomic, proteomic, imaging, and functional data to predict how each patient would respond to possible therapeutic interventions [205]. Other algorithms are developed to implement realistic heart rhythms [206]. The objective of computational modeling is to seize all that we know about disorders and to generate improved treatments tailored to the conditions of individuals, which is usually referred to as ‘computational medicine’ [207]. Computational medicine is applied in a myriad of areas, such as cancer, diabetes, cardiology, neurology, and so on [208]. However, more additional advancements in translating these computational methods to the clinic are essential [209,210]. The forthcoming computational medicine will also have to integrate more commonly the use of artificial intelligence to rectify the algorithm mistakes in order to enhance the predictive model confidence [211]. The advances in artificial intelligence and precision medicine are posed to revolutionize health care [212].

## Data Availability

Not applicable.

## Abbreviations

The following abbreviations are used in this manuscript:

MV	mitral vavle
TV	tricuspid valve
AV	aortic valve
PV	pulmonary valve
CFD	computational fluid dynamics
ISO	international organization for standardization
FDA	food and drug administration
FSI	fluid–structure interaction
IB	immersed boundary
FEM	finite-element method
2D	two-dimensional
3D	three-dimensional
CT	computed tomography
MRI	magnetic resonance imaging
ALE	arbitrary Lagrangian–Eulerian
SPH	smoothed-particle hydrodynamics
GPU	graphics processing unit
